# Reduced binaural masking release in women with migraine despite normal hearing: a case-control study

**DOI:** 10.3389/fnins.2026.1876406

**Published:** 2026-07-21

**Authors:** Ahmet Adnan Cırık, Yeşim Esen Yiğit Koçer, Fatih Mutlu, Reza Doğan, Sara Gül Avcı, Mustafa Men

**Affiliations:** 1 Department of Otolaryngology, Umraniye Training and Research Hospital, Istanbul, Türkiye; 2 Department of Otolaryngology, Kocaeli University, Kocaeli, Türkiye; 3 Department of Audiology, Umraniye Training and Research Hospital, Istanbul, Türkiye; 4 Department of Neurology, Umraniye Training and Research Hospital, Istanbul, Türkiye

**Keywords:** auditory brainstem, binaural interaction, central auditory processing, masking level difference, migraine

## Abstract

**Introduction:**

Migraine is a neurological disorder associated with altered sensory processing, including auditory hypersensitivity. Although peripheral hearing is typically normal, central auditory mechanisms—particularly binaural interaction—may be affected. The masking level difference (MLD) test evaluates binaural processing at the level of the auditory brainstem.

**Methods:**

This case-control study included 30 female patients with migraine and 35 age-matched healthy controls. All participants had normal otoscopic findings, type A tympanograms, and normal pure-tone thresholds. MLD testing was performed at 500 Hz. and 60 dB under homophasic (S_0_N_0_) and antiphasic (SπN_0_, S_0_Nπ) conditions. MLD values were calculated as the difference between homophasic and antiphasic thresholds. Group comparisons and ANCOVA were conducted.

**Results:**

Pure-tone thresholds were within normal limits but significantly higher in the migraine group (*p* < 0.001). No significant difference was observed in S_0_N_0_ thresholds. However, SπN_0_ and S_0_Nπ thresholds were significantly elevated in patients (*p* < 0.001). Accordingly, both MLD indices (S_0_N_0_–SπN_0_ and S_0_N_0_–S_0_Nπ) were significantly reduced in the migraine group (*p* < 0.001), and these differences remained significant after controlling for pure-tone averages.

**Conclusion:**

Migraine patients demonstrate reduced binaural masking release despite normal peripheral hearing, suggesting impaired brainstem-level binaural processing. These findings support altered central auditory function in migraine and may reflect broader subcortical sensory dysfunction.

## 1 Introduction

Migraine is a common neurological disorder characterized by recurrent episodes of pulsating headache and a variety of sensory symptoms. In addition to photophobia and phonophobia, many patients experience vestibular complaints such as dizziness or imbalance, which may occur during or between headache attacks. These manifestations are thought to reflect the involvement of multiple sensory pathways within the central nervous system, including auditory and vestibular circuits (Benjamin et al., 2022).

Although pure-tone audiometry is typically normal in individuals with migraine, increasing evidence indicates that auditory processing may be affected at suprathreshold levels. Patients often report sound hypersensitivity and reduced auditory tolerance, supporting the involvement of central auditory mechanisms (Goldstein and Stephens, 1975; Agessi et al., 2014). Previous studies evaluating auditory function in migraine have yielded inconsistent findings. Electrophysiological investigations using auditory brainstem response (ABR) have demonstrated alterations suggestive of brainstem involvement (Younis et al., 2019; Santiago et al., 2020), whereas behavioral central auditory processing (CAP) tests have shown variable results across different paradigms (Agessi et al., 2014; Cekic et al., 2024). These discrepancies indicate that migraine-related auditory dysfunction may not uniformly affect all aspects of central auditory processing.

Binaural interaction represents a specific auditory function that has received relatively limited attention in this population. The masking level difference (MLD) is a well-established measure of binaural processing that reflects phase-sensitive auditory mechanisms primarily at the level of the brainstem (Firat et al., 2006; Takeuti et al., 2019; Ignatious et al., 2023). Despite its relevance, studies investigating MLD in migraine remain scarce, particularly in individuals with vestibular migraine symptoms (Younis et al., 2019; Ali et al., 2025).

Therefore, the present study aims to evaluate binaural interaction using the MLD paradigm in adults with migraine symptoms compared to healthy controls. This study seeks to clarify whether phase-based binaural processing is altered in migraine-spectrum disorders and to contribute to the limited evidence in this area.

## 2 Materials and methods

This case-control study was conducted at a tertiary care hospital. Adult participants aged 18 to 65 years were recruited from the otolaryngology and neurology outpatient clinics. Inclusion criteria were normal otoscopic findings, type A tympanograms, and pure-tone audiometric thresholds within normal limits across all tested frequencies. Individuals with a history of chronic otitis media, previous ear surgery, hearing loss, unexplained dizziness, or any neurological disorder other than migraine were excluded. The patient group consisted of 30 individuals diagnosed with migraine based on clinical evaluation according to the International Classification of Headache Disorders, 3rd edition (ICHD-3) criteria [Headache Classification Committee of the International Headache Society (IHS), 2018]. The control group included 35 age-matched healthy adults with no history of migraine, vestibular complaints, neurological disease, or otologic pathology. Ethical approval was obtained from Ümraniye Training and Research Hospital on January 15, 2026, with decision number 482.

All subjects underwent standard pure-tone audiometric evaluation in a sound-treated booth. All audiological and psychoacoustic assessments were performed during the interictal period, at least 48–72 h after the last migraine attack, to minimize the possible influence of ictal or postictal auditory sensitivity.

Masking level difference (MLD) testing and pure-tone audiometric evaluation performed in a sound-treated room using an Interacoustics AC40 Hybrid clinical audiometer (Interacoustics A/S, Middelfart, Denmark) with calibrated supra-aural headphones. A 500-Hz tonal stimulus was presented in 60 dB narrow-band noise under three binaural phase conditions: S_0_N_0_, SπN_0_, and S_0_Nπ. In the S0N0 condition, both the signal and noise were presented in phase; in the SπN_0_ condition, the signal was presented in opposite phase while the noise remained in phase; and in the S_0_Nπ condition, the signal remained in phase while the noise was presented in opposite phase. The presentation level of the stimulus was varied to determine the detection threshold for each condition. The order of test conditions was randomized to minimize order effects. MLD values were calculated as the differences between the homophasic and antiphasic masked thresholds using the formulas S_0_N_0_–SπN_0_ and S_0_N_0_–S_0_Nπ. All measurements were performed by the same experienced audiologist, and the audiometer was calibrated before data collection.

Demographic variables and audiometric outcomes were recorded for statistical analysis. Between-group comparisons of pure-tone thresholds and MLD values were performed between the migraine and healthy control groups. Data normality was assessed before analysis, and independent-samples *t*-tests or Mann-Whitney *U* tests were used according to the distribution of the variables.

To determine whether group differences in MLD threshold values were independent of pure-tone hearing levels, ANCOVA was performed with group as the fixed factor and right and left pure-tone averages (PTA) entered as covariates. The assumptions of homogeneity of variances and homogeneity of regression slopes were assessed using Levene's test and the group × covariate interaction term, respectively. Effect size was reported as partial eta squared (η^2^), and statistical significance was set at *p* < 0.05.

## 3 Results

All participants were female. The overall mean age was 32.4 ± 7.6 years. The mean age was 34.0 ± 8.6 years in the migraine group and 31.0 ± 6.3 years in the control group. Ages ranged from 20 to 49 years in the migraine group and from 23 to 52 years in the control group. There was no statistically significant difference between the groups in terms of mean age, indicating that the groups were comparable with respect to age.

Among the patients with migraine (*n* = 30), unilateral headache localization was observed in most cases (76.7%), and left hemicranial pain was the most common localization pattern (40.0%). The most frequently reported accompanying symptoms were photophobia (90.0%) and phonophobia (86.7%), followed by nausea (56.7%). Visual aura (10.0%), tinnitus (6.7%), and facial numbness (3.3%) were observed less frequently (Table 1).

**Table 1:** Distribution of photophobia, phonophobia, and all other symptoms in the migraine group.

Clinical characteristic	Category	Migraine patients	
		*n*	%
Location of the pain	Bilateral	7	23.3
	Right	11	36.7
	Left	12	40.0
Sonophobia	Absent	4	13.3
	Present	26	86.7
Photophobia	Absent	3	10.0
	Present	27	90.0
Visual aura	Absent	27	90.0
	Present	3	10.0
Numbness in face	Absent	29	96.7
	Present	1	3.3
Nausea	Absent	13	43.3
	Present	17	56.7
Tinnitus	Absent	28	93.3
	Present	2	6.7

When pure-tone audiometry (PTA) averages were compared between the migraine and control groups, hearing thresholds were within normal limits in both groups; however, mean PTA values were significantly higher in the migraine group for both ears. The right-ear PTA was 11.45 ± 3.98 dB HL in the migraine group and 7.04 ± 3.18 dB HL in the control group (*t* = 4.97, *p* < 0.001). The left-ear PTA was 10.81 ± 3.92 dB HL in the migraine group and 6.80 ± 3.20 dB HL in the control group (*t* = 4.55, *p* < 0.001) (Table 2).

**Table 2:** Comparison of pure-tone audiometry findings between the migraine and control groups.

Parameter	Migraine (*n* = 30)	Normal (*n* = 35)	*p*
Right PTA			
Mean ± SD	11.45 ± 3.98	7.04 ± 3.18	< 0.001^a***^
Median (IQR)	11.43 (4.11)	7.14 (3.93)	
Left PTA			
Mean ± SD	10.81 ± 3.92	6.80 ± 3.20	< 0.001^a***^
Median (IQR)	10.71 (6.96)	6.43 (3.57)	

^a^Student-*t* Testi. ^***^p < 0.001. PTA, Pure tone audiometry; IQR, Interquartile range.

Comparison of acoustic measurement results between the groups revealed no statistically significant difference in the S_0_N_0_ parameter (*p* = 0.112). In contrast, the SπN_0_ and S_0_Nπ parameters were significantly higher in the migraine group than in the control group (both *p* < 0.001) (Table 3).

**Table 3:** Comparison of MLD thresholds between the migraine and control groups.

Binaural phase condition	Description	Normal	Migraine	*p*
S_0_N_0_	Avg ± SD	60.89 ± 2.36	61.67 ± 3.93	0.112^b^
	Median (Min–Max)	62 (56–66)	62 (48–68)	
SπN_0_	Avg ± SD	50.97 ± 3.49	55.4 ± 5.07	< 0.001^a^
	Median (Min–Max)	50 (40–58)	54 (46–66)	
S_0_Nπ	Avg ± SD	47.51 ± 3.52	53.6 ± 4.58	< 0.001^a^
	Median (Min–Max)	48 (38–54)	52 (46–64)	

^a^Independent *t* test; ^b^Mann-Whitney *U* test. S_0_N_0_: (Homophasic condition) the signal and noise are presented in phase to both ears. SπN_0_: (Antiphasic signal condition) the signal is presented 180° out of phase between the ears, while the noise remains in phase. S_0_Nπ: (Antiphasic noise condition) the signal is presented in phase, while the noise is presented 180° out of phase between the ears.

**Figure 1:**
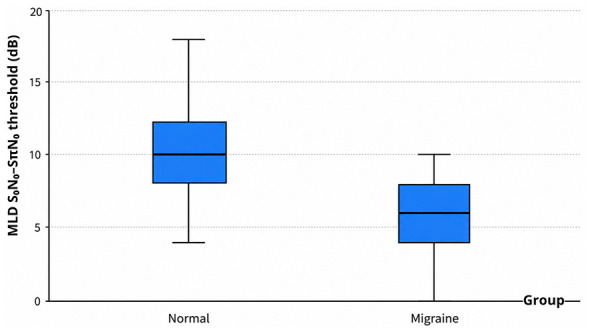
Comparison of MLD S_0_N_0_–SπN_0_ of the two groups.

**Figure 2:**
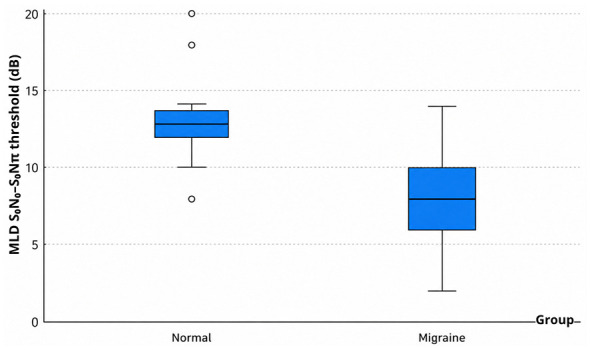
Comparison of MLD S_0_N_0_–S_0_Nπ of the two groups.

Evaluation of the acoustic difference parameters showed that both MLD values, calculated as S_0_N0–SπN_0_ and S_0_N_0_–S_0_Nπ, were significantly lower in the migraine group than in the control group (both *p* < 0.001) (Table 4 and Figures 1, 2).

**Table 4:** Comparison of MLD values.

MLD condition	Description	Normal	Migraine	*p*
MLD: S_0_N_0_–SπN_0_	Avg ± SD	9.91 ± 2.74	6.27 ± 3.1	< 0.001^a^
	Median (Min–Max)	10 (4–18)	6 (0–10)	
MLD: S_0_N_0_–S_0_Nπ	Avg ± SD	13.37 ± 2.5	8.07 ± 3.45	< 0.001^b^
	Median (Min–Max)	13 (8–20)	8 (2–14)	

^a^Independent *t* test; ^b^Mann-Whitney *U* test.

MLD, Masking level difference. S_0_N_0_: (Homophasic condition) the signal and noise are presented in phase to both ears. SπN_0_: (Antiphasic signal condition) the signal is presented 180° out of phase between the ears, while the noise remains in phase. S_0_Nπ: (Antiphasic noise condition) the signal is presented in phase, while the noise is presented 180° out of phase between the ears.

**Figure 3:**
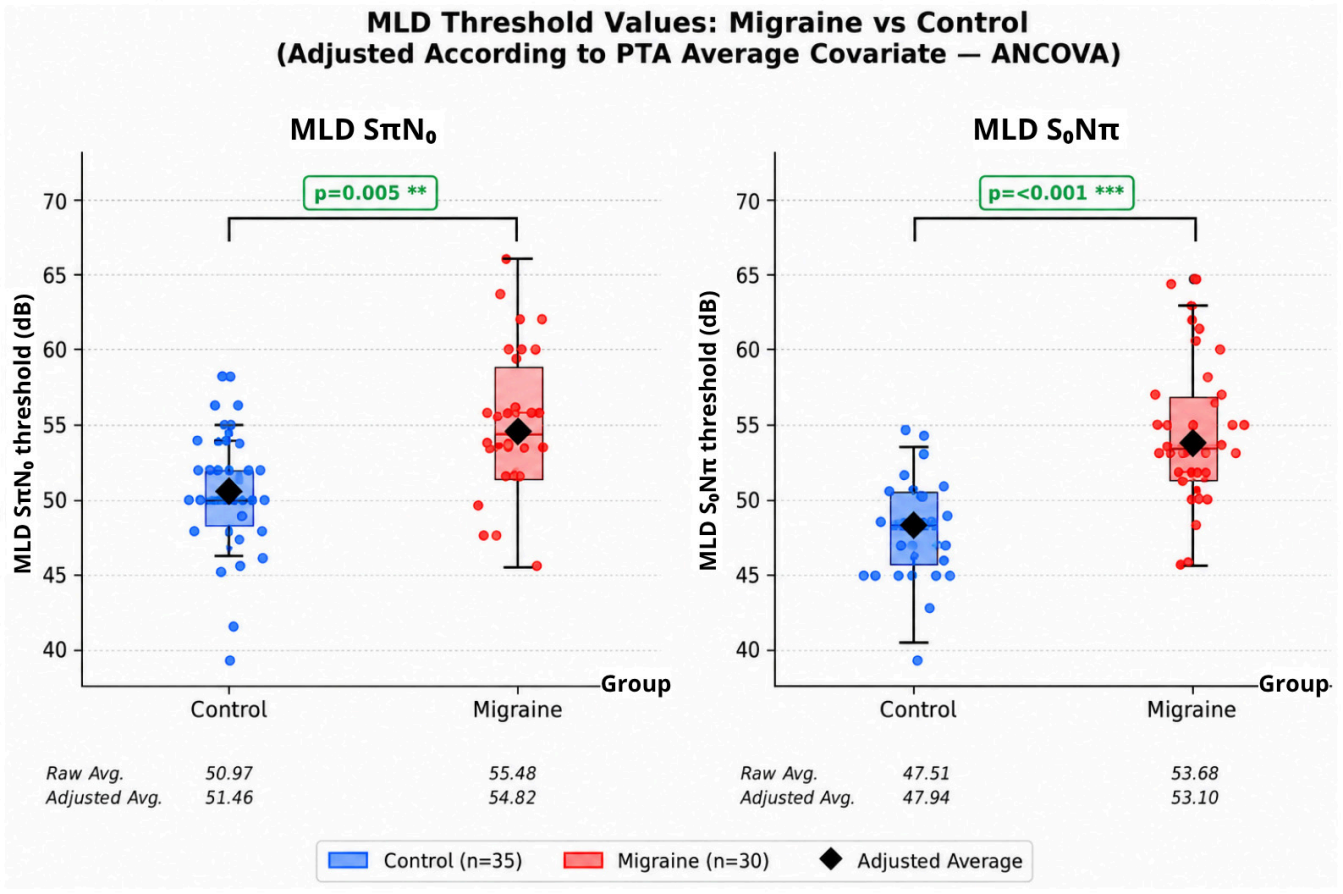
Distribution of MLD SπN_0_ and MLD S_0_Nπ thresholds in the migraine and control groups after adjustment according to PTA Average Covariate- ANCOVA [Boxes: interquartile range (IQR); central lines: medians; whiskers: 1.5 × IQR; individual points: raw measurements; diamonds: ANCOVA-adjusted values].

After adjustment for right and left pure-tone averages (PTA) using ANCOVA, both MLD SπN_0_ and MLD S_0_Nπ threshold values were significantly higher in the migraine group than in the control group. The adjusted mean MLD SπN_0_ threshold was 54.82 dB in the migraine group and 51.46 dB in the control group (*F* = 8.66, *p* = 0.005, η^2^ = 0,124). Similarly, the adjusted mean MLD S_0_Nπ threshold was higher in the migraine group than in the control group, with values of 53.10 dB and 47.94 dB, respectively (*F* = 22.81, *p* < 0.001, η^2^ = 0.272) (Tables 5, 6 and Figure 3).

**Table 5:** MLD threshold values: comparison of the two groups (ANCOVA).

Parameter	Migraine (*n* = 30) Avg ±Std Median (IQR)	Control (*n* = 35) Avg ±Std Median (IQR)	Migraine Adjusted means^a^ (dB)	Control Adjusted means^a^ (dB)	*F*	*p*	η^2^
MLD Sπ*N*_0_	55.40 ± 5.07	50.97 ± 3.49	54.82	51.46	8.66	0.005^**^	0.124
	54.00 (6.50)	50.00 (2.00)					
MLD S_0_*Nπ*	53.60 ± 4.58	47.51 ± 3.52	53.10	47.94	22.81	<0.001^***^	0.272
	52.00 (4.50)	48.00 (4.50)					

^a^Adjusted means were calculated by setting the covariates equal to their grand means (Right ear PTA = 8.13 dB, Left ear PTA = 8.04 dB). ^**^p < 0,01, ^***^p < 0.001 | η^2^: partial eta squared (effect size).

MLD, Masking level difference. SπN_0_: (Antiphasic signal condition) the signal is presented 180° out of phase between the ears, while the noise remains in phase. S_0_Nπ: (Antiphasic noise condition) the signal is presented in phase, while the noise is presented 180° out of phase between the ears.

**Table 6:** ANCOVA assumption tests.

Parameter	Levene *p*	Slope homogeneity		Covariate	
	Homogeneity of variance	DIAGNOSIS × Right PTA *p*	DIAGNOSIS × Left PTA *p*	Right PTA *p*	Left PTA *p*
MLD Sπ*N*_0_	0.087 ✓	0.403 ✓	0.391 ✓	0.214 ✓	0.810 ✓
MLD S_0_*Nπ*	0.233 ✓	0.075 ✓	0.861 ✓	0.153 ✓	0.919 ✓

MLD, Masking level difference. SπN0: (Antiphasic signal condition) the signal is presented 180° out of phase between the ears, while the noise remains in phase. S_0_Nπ: (Antiphasic noise condition) the signal is presented in phase, while the noise is presented 180° out of phase between the ears. PTA, Pure tone average.

Distribution of MLD SπN_0_ and MLD S_0_Nπ thresholds across the migraine and control groups. Box: IQR; central line: median; whiskers: 1.5 × IQR; points: individual measurements; diamond: group mean adjusted after controlling for SSO as a covariate.

## 4 Discussion

The present study demonstrated that women with migraine showed significantly reduced masking level difference (MLD) values despite preserved S_0_N_0_ thresholds. In brief, this indicates that while basic signal detection in noise remained intact, the additional binaural advantage normally obtained through interaural phase disparity was significantly diminished in the migraine group.

MLD is a behavioral measure of binaural interaction and binaural masking release, reflecting the auditory system's ability to use interaural phase differences to improve signal detection in noise. Neurophysiologically, this mechanism is primarily mediated at the level of the auditory brainstem, particularly within the superior olivary complex and lower brainstem binaural pathways, where phase-sensitive comparison of bilateral auditory input occurs. Therefore, reduced MLD values in the migraine group may indicate impaired brainstem-level binaural integration mechanisms rather than peripheral cochlear dysfunction (Santiago et al., 2020).

Given that migraine is increasingly recognized as a disorder characterized by altered subcortical sensory network excitability and abnormal neurophysiological responses across multiple sensory modalities (Younis et al., 2019; Ulutas et al., 2025), the observed reduction in MLD may indicate dysfunction not only in brainstem-level binaural interaction but also in broader subcortical auditory processing networks.

Migraine has been conceptualized as a disorder involving altered sensory processing within central networks, including subcortical structures. Electrophysiological studies using auditory brainstem response (ABR) have reported alterations in wave latencies and interpeak intervals in patients with migraine, suggesting possible brainstem involvement (Firat et al., 2006). Similarly, controlled studies in women with vestibular migraine have demonstrated electrophysiological patterns consistent with altered auditory brainstem function (Takeuti et al., 2019).

Although both ABR and MLD are related to brainstem function, they assess different aspects of auditory processing. ABR is an objective measure of neural conduction and synchrony along the auditory pathway, whereas MLD is a behavioral measure of functional binaural integration under masking conditions. Thus, the present findings complement electrophysiological evidence by demonstrating that functional binaural unmasking may be reduced even when standard audiometric thresholds remain within normal limits. A recent case-control study combining ABR and MLD measures in patients with migraine similarly reported evidence of altered auditory brainstem-related processing despite normal peripheral hearing (Ali et al., 2025). Although methodological details and sample characteristics differed, the convergence of MLD and electrophysiological findings strengthens the hypothesis that migraine may affect brainstem-level auditory mechanisms beyond what can be detected using conventional audiometry.

It is also noteworthy that behavioral central auditory processing studies in migraine have yielded heterogeneous findings depending on the specific test paradigm employed. For example, a study using the Duration Pattern Test and Frequency Pattern Test did not identify significant overall group differences, although age and sex were found to influence performance in certain domains (Yeral et al., 2024). This variability suggests that migraine-related auditory alterations may not uniformly affect all central auditory tasks, but may instead selectively affect mechanisms such as binaural phase-based processing. In this context, the present findings suggest that MLD paradigms may be particularly sensitive to subtle differences in binaural interaction in patients with migraine.

Clinically, the high prevalence of photophobia and phonophobia in the migraine group is consistent with the broader sensory hypersensitivity phenotype characteristic of migraine. Reduced binaural masking release may contribute to everyday listening difficulties, particularly in complex acoustic environments where the effective use of interaural cues enhances signal detection.

Although the inclusion of an exclusively female sample reduced sex-related heterogeneity, it limits the generalizability of the findings to male or mixed-sex populations; therefore, the interpretation of MLD magnitude should consider sex-specific normative data. Recent normative findings in healthy young adults have shown that MLD values may vary according to sex and testing parameters, emphasizing the importance of appropriate reference data in clinical interpretation (Cekic et al., 2024). In the present study, however, the significant between-group differences in both MLD indices within a uniform female sample suggest that the observed reduction is unlikely to be attributable solely to sex-related variability.

While all participants included in the present study had pure-tone audiometric thresholds within normal limits (< 15 dB HL), mean hearing thresholds differed significantly between the migraine and control groups. Nevertheless, after adjustment for right- and left-ear pure-tone average values using ANCOVA, MLD thresholds remained significantly higher in the migraine group than in the control group.

This study is subject to several limitations. During the study period, the eligible patient pool consisted only of female patients. Therefore, the study population composed solely of female participants. While this provided sex-related homogeneity, it also restricted the generalizability of the findings to mixed-sex populations. Additionally, a larger sample size would have allowed subgroup analyses among patients with migraine and may have provided further insight into the potential influence of clinical features, such as aura, on the study outcomes. Furthermore, as the MLD is a behavioral measure, the possible influence of attentional and cognitive factors cannot be entirely excluded. Future studies combining behavioral MLD paradigms with electrophysiological measures such as ABR may further clarify the relationship between perceptual binaural integration and neural conduction abnormalities.

## 5 Conclusions

Patients with migraine showed preserved homophasic masked thresholds but significantly reduced binaural masking release, as reflected by lower S_0_N_0_–SπN_0_ and S_0_N_0_–S_0_Nπ differences. These findings suggest altered binaural interaction mechanisms, potentially associated with brainstem-level auditory processing abnormalities in migraine, even in the presence of normal peripheral hearing.

## Data Availability

The raw data supporting the conclusions of this article will be made available by the authors, without undue reservation.
